# Effect of the Chemical Properties of Silane Coupling Agents on Interfacial Bonding Strength with Thermoplastics in the Resizing of Recycled Carbon Fibers

**DOI:** 10.3390/polym15214273

**Published:** 2023-10-30

**Authors:** Hyunkyung Lee, Minsu Kim, Gyungha Kim, Daeup Kim

**Affiliations:** 1 Carbon & Light Materials Application Group, Korea Institute of Industrial Technology, Bucheon 14449, Republic of Korea; dori9424@gmail.com (H.L.); mskim85@kitech.re.kr (M.K.); 2 Department of Carbon Material Fiber Engineering, Chonbuk National University, Jeonju 54896, Republic of Korea

**Keywords:** carbon fiber, resizing, silane coupling agent, thermoplastic, interfacial shear strength, mechanism, oxygen functional group

## Abstract

Upcycling recycled carbon fibers recovered from waste carbon composites can reduce the price of carbon fibers while improving disposal-related environmental problems. This study assessed and characterized recycled carbon fibers subjected to sizing treatment using N-(2-aminoethyl)-3-aminopropyltrimethoxysilane (APS) chemically coordinated with polyamide 6 (PA6) and polypropylene (PP) resins. Sizing treatment with 1 wt.% APS for 10 s yielded O=C-O on the surface of the carbon fiber, and the -SiOH in the APS underwent a dehydration–condensation reaction that converted O=C-O (lactone groups) into bonds of C-O (hydroxyl groups) and C=O (carbonyl groups). The effects of C-O and C=O on the interfacial bonding force increased to a maximum, resulting in an oxygen-to-carbon ratio (O/C) of 0.26. The polar/surface energy ratio showed the highest value of 32.29% at 10 s, and the interfacial bonding force showed the maximum value of 32 MPa at 10 s, which is about 15% better than that of commercial carbon fiber (PA6-based condition). In 10 s resizing treatments with 0.5 wt.% 3-methacryloxypropyltrimethoxysilane (MPS), C-O, C=O, and O=C-O underwent a dehydration–condensation reaction with -SiOH, which broke the bonds between carbon and oxygen and introduced a methacrylate group (H2C=C(CH_3_)CO_2_H), resulting in a significant increase in C-O and C=O, with an O/C of 0.51. The polar/surface free energy ratio was about 38% at 10 s, with the interfacial bonding force increasing to 27% compared to commercial carbon fiber (PP-based conditions). MPS exhibited a superior interfacial shear strength improvement, two times higher than that of APS, with excellent coordination with PP resin and commercial carbon fiber, although the interfacial bonding strength of the PP resin was significantly lower.

## 1. Introduction

Carbon fibers are lightweight materials with low density, high specific strength, heat resistance, and excellent thermal and electrical conductivity, and their applications are expected to expand not only in the aerospace industry but also in all industries in the future [1,2,3,4,5,6,7,8,9]. However, due to the high price of carbon fiber and the expensive manufacturing process, it is only used for expensive parts, such as aerospace, shipbuilding, and sporting goods, and it is difficult to expand its application to fields such as general commercial vehicles due to its high price [10,11]. In addition, carbon composites currently in use are made of thermosetting resins, which are difficult to recycle, and most are disposed of by landfill and incineration, causing environmental pollution [12]. To reduce the price of carbon fibers and solve environmental pollution problems, upcycling technology to recover waste carbon composites for recycling is absolutely necessary [13,14,15,16].

The surface properties of carbon fibers greatly affect the mechanical properties of carbon composites, and surface treatment and sizing are essential for upcycling recycled carbon fibers recovered from previously used carbon composites to achieve properties equivalent to commercial carbon fiber without degradation [17]. Sizing treatment is a simple process that protects the surface by coating the carbon fiber with an interfacial binder, while offering a stable interface by improving the chemical bonding force with the resin to yield better chemical and mechanical properties than those obtained by surface treatment of general carbon fibers [18,19,20]. Sizing treatments include coating with organic polymers and coating with metal oxides, which are inorganic molecules, and converting them into metal crystals to form a protective film [19,20]. The sizing treatment of carbon fibers with poly(phthalazinone ether ketone) was previously shown to result in C-N and C=N bonds present in the phthalazine ring, which improved the thermal stability, and the surface energy was enhanced by the increase in C=O bonds [21]. 

The coating of basalt fibers with an amino–silane coupling agent reportedly enhanced the interfacial bonding of basalt fibers and PA66 by the non-polar CH_2_ chains and polar amino groups of the silane coupling agent. As the number of CH_2_ chains increased, the chain entanglement between Si molecules and PA66 improved the interfacial bonding [22]. By coating the basalt felt (BF) surface with a nickel-based metal-organic framework (Ni-MOF), the papers reported that the weak interfacial bond with the epoxy resin was improved by the self-lubricating behavior of Ni-MOF during friction, increasing the interfacial bond strength by about 15.19%. [23]. Changes in the chemical properties of carbon fibers sized with E51 epoxy resin and the curing agent DDS, analyzed using X-ray photoelectron spectroscopy (XPS), showed enhanced epoxide bond formation and interfacial shear strength (IFSS), which were not seen in untreated carbon fibers [5]. Further, sizing treatment with 4,4′-diphenylmethane diisocyanate can increase the content of oxygen functional groups via the chemical reaction of carbonyl, carboxyl, and -NCO groups on the carbon fiber surface and can improve the wettability between carbon fiber and resin [24]. 

Carbon fibers treated with poly(amidoamine) can improve the mechanical properties of carbon composites by forming covalent bonds between amino groups and epoxy resin [25], and carbon fibers sized with carboxylic polyphenylene sulfide (PPS-COOH) have been shown to increase interfacial bonding with polyphenylene sulfide resin by eliminating C-N bonds, forming new C-S bonds, and increasing the content of C=O [26]. According to research so far, most studies have been conducted on the mechanical and chemical properties according to the sizing type and processing conditions of commercial carbon fiber and resin; however, the mechanism of surface chemical structure changes during resizing treatment using recycled carbon fibers has not been clarified.

In this study, to improve the interfacial bonding between recycled carbon fibers and resins for the purpose of upcycling recycled carbon fibers, recycled carbon fibers were desized and then resized using silane coupling agents that are chemically compatible with thermoplastic PA6 and PP resins. This study aimed to identify the optimal conditions under which recycled carbon fibers with resizing treatment have the same physical and chemical properties as commercial carbon fiber and to investigate the effects and mechanisms of PA6 and PP silane coupling agents on the interfacial bonding force between recycled carbon fibers and thermoplastic resins.

## 2. Materials and Methods

### 2.1. Materials

**Figure 1:**
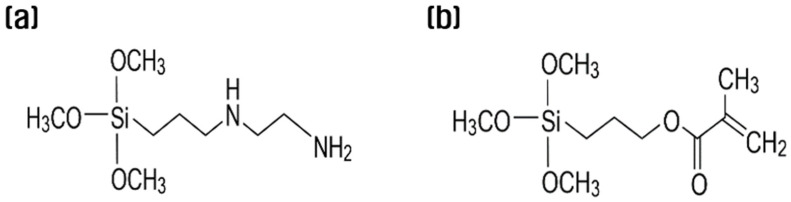
Chemical formula of (**a**) N-(2-aminoethyl)-3-aminopropyltrimethoxysilane and (**b**) 3-methacryloxypropyltrimethoxysilane.

This study used recycled carbon fibers recovered from hydrogen tanks, and their physical properties were compared with Toray’s commercial carbon fiber, as shown in Table 1. In accordance with the ASTM D3822 standard [27], tensile tests were conducted at a tensile speed of 5 mm/min, and the average value was calculated for the results of >20 tests per condition. The silane coupling agents used in the resizing process were N-(2-aminoethyl)-3-aminopropyltrimethoxy silane (KBM-602, 99.9%, Shin Etsu, Tokyo, Japan, hereinafter referred to as APS), which has good chemical harmony with PA6 (Figure 1a), and 3-methacryloxypropyltrimethoxysilane (KBM-503, 99.9%, Shin Etsu, Tokyo, Japan, hereinafter referred to as MPS), which has good chemical harmony with PP (Figure 1b). The chemical structures of the sizing agents used in this study are shown in Figure 1.

### 2.2. Experimental Methods

To desize recycled carbon fibers, they were treated in acetone (99.5%, Daejung Chemical, Siheung-si, Republic of Korea) at 60 °C for 30 min to completely remove the chemical components remaining on the surface of the carbon fibers when they were recovered and separated from the waste carbon composite. Surface treatment was then performed by immersion in nitric acid (60.0%, Samchun Pure Chemical, Seoul, Republic of Korea) at 100 °C for 1 h, and the carbon fiber without surface treatment and resizing was labeled “untreated.” Recycled carbon fiber with nitric acid surface treatment was subjected to resizing treatment, and the sizing agent was prepared by adding 0.5–2 wt.% silane coupling agent to ethanol (99.5%, Daejung Chemical, Siheung-si, Republic of Korea) and distilled water. The recycled carbon fibers with nitric acid surface treatment were immersed in the sizing agent for 3–15 s for resizing and then dried at 120 °C for 2 h.

### 2.3. Characteristic Analysis

The recycled carbon fiber with the resizing treatment was analyzed for the amount of sizing agent coated on the surface of the recycled carbon fiber using thermogravimetric analysis (TGA, WATERS (TA Instruments, New Castle, DE, USA) (Discovery SDT 650)), and the sample was heated up to 1000 °C at a ramping rate of 10 °C/min in a nitrogen atmosphere to analyze the change in thermal weight. IFSS (Interfacial shear strength tester, ST-1000) was performed based on ASTM C1557 [28] to analyze the interfacial bonding strength between carbon fiber and resin; the interfacial shear strength was evaluated through the pull-out method by depositing 200 μm of carbon fiber into the resin and pulling it out at a speed of 0.1 mm/min, and the average value was used for 25 tests per test condition.

XPS from Nexsa (Thermo Fisher Scientific Inc., Whaltman, MA, USA) was used to investigate the changes in the chemical functional groups on the surface of recycled carbon fibers following resizing treatment. The specimens were irradiated with monochromatic Al Kα (1486.6 eV), and high-resolution spectra were obtained at a pass energy of 50 eV and a beam size of 400 μm. In addition, to analyze the surface energy changes, the contact angle of each condition was measured using the Wilhelmy Plate Method based on ASTM D1331-20 [29], in which the carbon fiber was dropped into hydrophilic water and hydrophobic diiodomethane (99.9%, Sigma–Aldrich Co., Llc., St. Louis, MO, USA) at a constant injection rate of 6 mm/min. The contact angle measurements were evaluated five times per condition, and the surface energy was calculated from the contact angle obtained from the angle of immersion of the carbon fiber in the sample and its exit angle.

## 3. Results and Discussion

### 3.1. Thermal Properties of Recycled Carbon Fibers

**Figure 2:**
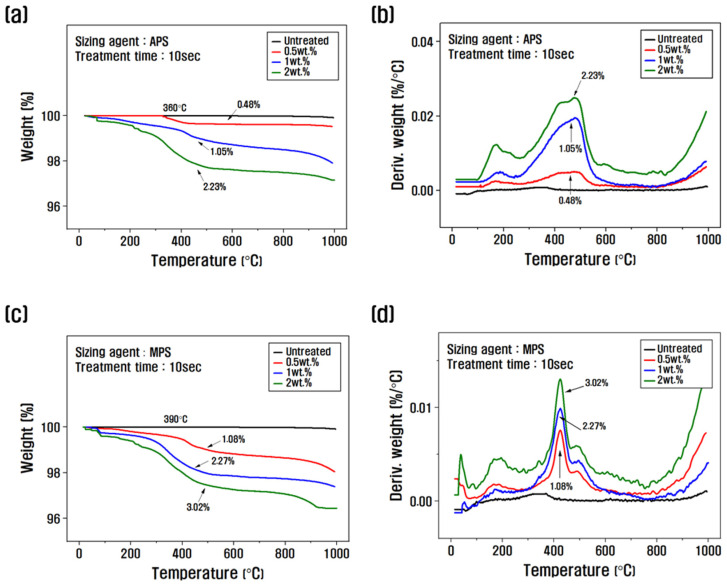

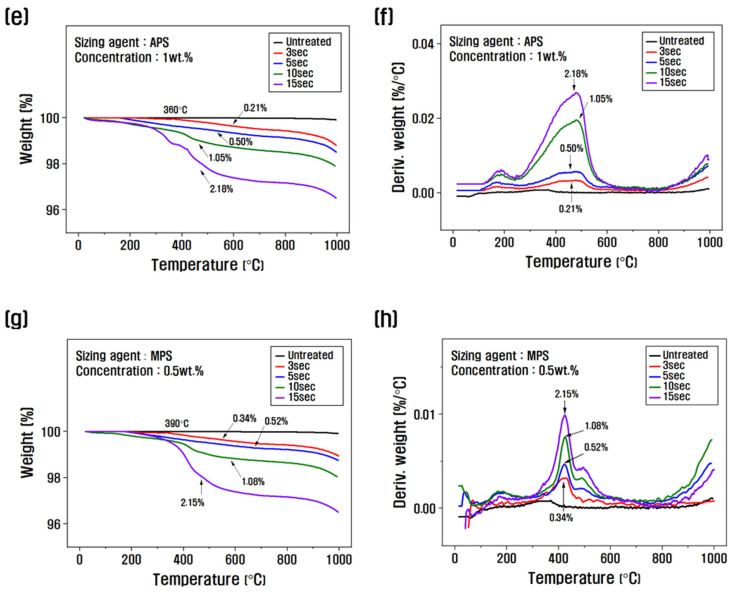
Thermogravimetric analysis and derivative thermogravimetry of recycled carbon fibers according to the concentration and treatment time of the silane coupling agent: (**a**,**b**,**e**,**f**) rCF/APS, (**c**,**d**,**g**,**h**) rCF/MPS.

The amount of sizing agent bound to the surface of the recycled carbon fiber was evaluated using TGA to assess changes in thermogravimetric weight. Figure 2 shows the TGA graph as a function of the sizing agent concentration and the resizing treatment time. To select the optimal concentration, the treatment time was fixed at 10 s, and the resizing was performed according to the change in concentration from 0.5 to 2 wt.% (Figure 2a–d). The weight loss of the PA6-based APS sizing agent was about 0.48% at a concentration of 0.5 wt.%, about 1.05% when resizing was performed at 1 wt.% and about 2.23% for a concentration of 2 wt.%. The weight loss of the PP-based MPS sizing agent was about 1.08% at a concentration of 0.5 wt.% and about 2.27% at a concentration of 1 wt.%, which was significantly higher than that of APS. Regarding silane coupling agents, the best properties are reportedly obtained when the carbon fibers are coated with 1% of the agent. When sizing agents with a concentration of 1 wt.% or more are used, the concentration of silane-based substances that exhibit stiff properties should be gradually increased to minimize the impact on the properties [24]. Therefore, in this study, the concentration of 1 wt.% for APS and 0.5 wt.% for MPS was optimally fixed as a coating condition of 1% from the TGA results, and the changes with treatment time were evaluated. 

When fixing the APS concentration at 1 wt.% and observing the weight loss as a function of treatment time, the weight change was about 0.21% at 3 s, about 0.5% at 5 s, about 1.05% at 10 s, and about 2.18% at 15 s, and the weight loss gradually increased (Figure 2e,f). When the MPS concentration was fixed at 0.5 wt.% and the weight change with treatment time was analyzed, the weight change was about 0.34% and about 0.52% for the resizing treatment for 3 s and 5 s, respectively, about 1.08% at 10 s, and about 2.15% at 15 s (Figure 2g,h). This is believed to be due to the larger molecular chain and molecular weight of MPS compared to APS, which results in a faster coating of the recycled carbon fiber. According to the TGA graph, the weight loss in the range of 100–200 °C was caused by the evaporation of water present in the recycled carbon fiber, and the weight change after 300 °C was due to the removal of water molecules in the -SiOH condensation reaction on the surface of the recycled carbon fiber and the thermal degradation of the silane coupling agent [30,31]. 

Other studies have reported that when more than 1% sizing treatment is applied to the surface of carbon fibers, fiber-to-fiber bonding and agglomeration are observed. This phenomenon causes the deterioration of the mechanical properties of carbon composites, due to the difficulty of the resin in penetrating between the carbon fibers when mixed to produce carbon composites, and becomes more severe as the coating amount of the sizing agent increases [21,32,33]. In this study, TGA results showed that a 1% sizing agent coating on the surface of recycled carbon fibers was the optimal condition. The PA6 sizing agent was optimal at a concentration of 1 wt.% and a treatment time of 10 s, whereas the PP sizing agent was optimal at a concentration of 0.5 wt.% and a treatment time of 10 s.

### 3.2. Mechanical Properties of Recycled Carbon Fibers

**Figure 3:**
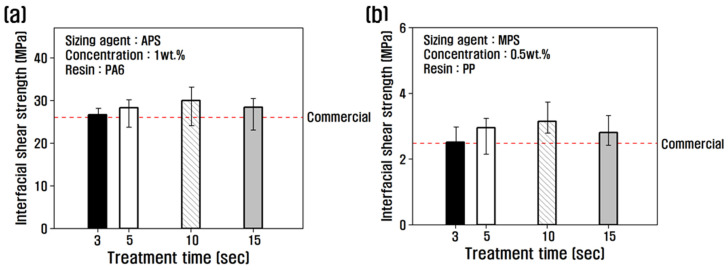
Interfacial shear stress for (**a**) rCF/APS and (**b**) rCF/MPS by single fiber pull-out testing.

The interfacial shear strength was evaluated as a function of treatment time at the optimum concentration, and the results are shown in Figure 3. The recycled carbon fibers treated with PA6-based APS sizing agent were compared with PA6 resin, and the recycled carbon fibers treated with PP-based MPS sizing agent were compared with PP resin. For the recycled carbon fibers treated with APS at a concentration of 1 wt.%, the interfacial shear strength increased with increasing treatment time, reaching a maximum of 32 MPa at 10 s, and began to decrease at 15 s (Figure 3a). For MPS at a concentration of 0.5 wt.%, the interfacial shear strength gradually increased until the treatment time of 10 s, and decreased by about 11% at 15 s, compared to 10 s. 

Na Sun et al. reported that when the amount of sizing agent coated is low, the microscopic grooves present on the surface during carbon fiber manufacturing are not completely filled, which leads to pores at the interface of PA6 resin and carbon fiber during composite formation and reduces the interfacial bonding force [33]. The sizing agent-coated layer can improve interfacial bonding through chemical bonding and intermolecular attraction by chain entanglement, thereby preventing cracks from occurring at the interface with the resin [26]. A study reported that the interfacial shear strength was improved due to better impregnation, a rough surface, and high surface free energy between carbon fiber and resin [21].

This study confirmed that the interfacial shear strength was improved, compared to commercial carbon fiber, by resizing treatment under optimal conditions. When treated with APS for 10 s, the interfacial shear strength increased by about 15% compared to commercial carbon fiber at a concentration of 1 wt.%, and it increased by about 27% when treated with MPS at a concentration of 0.5 wt.% for 10 s. This is attributed to the improvement of the interfacial bonding force through a more active chemical reaction between the carbon fiber and the resin with the use of a sizing agent which has better chemical coordination with each resin than the epoxy sizing agent coated on commercial carbon fiber. 

The rate of increase in the interfacial bonding force of carbon fibers treated with a non-polar PP-based MPS sizing agent, which has a significantly lower interfacial bonding force with carbon fiber and resin than the PA6-based APS sizing agent, was higher. This is attributed to the optimal surface treatment in this study and the resizing treatment with MPS, which has a good chemical bond with PP resin and contributes to an improved interfacial bonding force with carbon fiber than using epoxy, a sizing agent used on the surface of commercial carbon fiber. However, the interfacial shear strength decreased when the concentration of APS was 1 wt.% for more than 10 s and when MPS was 0.5 wt.% for more than 10 s. Previous studies have indicated that increased resizing treatment time can result in an uneven coating layer on the carbon fiber surface when the sizing agent is 1% or more, resulting in sizing agent agglomeration and the breakdown of the interfacial bonding in the sizing layer due to van der Waals interaction between sizing agent molecules, which results in a decrease in the interfacial shear strength [34].

### 3.3. Chemical Properties of Recycled Carbon Fibers

**Figure 4:**
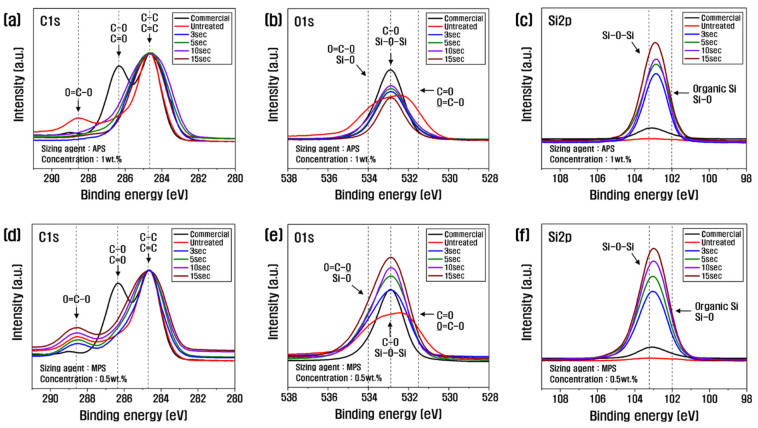
C1s and O1s XPS spectra of recycled carbon fibers covered with (**a**–**c**) APS and (**d**–**f**) MPS.

Figure 4 shows the C1s and O1s XPS spectra from the analysis of the chemical state of the recycled carbon fiber surface according to the resizing conditions. The oxygen-to-carbon ratio (O/C) for judging the degree of composition change and oxygen content increase is summarized in Table 2. With a 1 wt.% PA6-based APS sizing agent, the content of carbon and oxygen decreased, and the content of nitrogen and silicon increased, compared to the untreated control. The carbon content decreased until 10 s and increased at 15 s; oxygen showed the highest value at 10 s, and the nitrogen and silicon content increased continuously as the treatment time increased. By contrast, with the 0.5 wt.% PP-based MPS sizing agent, the amount of carbon decreased as the treatment time increased, and the amount of oxygen and silicon increased. The ratio of O/C, which indicates the degree of activity of the carbon fiber surface, showed the highest value of 0.26 at 10 s with APS treatment, and the recycled carbon fiber treated with MPS gradually increased as treatment time increased. The optimum value of 0.51 at 10 s was about twice that of the APS. Other studies have reported that the surface activity of carbon fibers is enhanced when the O/C is higher than 0.26 [11]. As shown in Table 2, the O/C of commercial carbon fiber is 0.28; thus, the recycled carbon fibers in this study are considered to have sufficiently introduced oxygen functional groups. Further, the reason for the significantly higher O/C of MPS compared to APS is thought to be the increased introduction of oxygen functional groups due to the large amount of oxygen contained in MPS.

According to the C1s spectra, the O=C-O (lactone group), which was present in the untreated carbon fiber without sizing treatment, decreased significantly as time increased, while the C-O (hydroxyl group) and C=O (carbonyl group) increased up to 10 s of treatment time and decreased at 15 s (Figure 4a, Table 3). This is because, up to 10 s, the -SiOH (silanol group) present in the APS reacted with the O=C-O on the surface of the recycled carbon fiber in a dehydration–condensation reaction, causing the O=C-O bond to decrease and the O connected to the O=C-O to bond with another carbon, resulting in an increase in C-O and C=O; however, beyond 10 s, the hydrolysis of APS is actively occurring, resulting in a decrease in O=C-O, C-O, and C=O [34]. However, after the resizing treatment with MPS, O=C-O, C-O, and C=O increased continuously with the increasing treatment time (Figure 4d, Table 3). This is possibly due to the -SiOH in MPS that reacted with the O=C-O in the recycled carbon fiber, breaking the bonds and increasing the amount of C-O and C=O. MPS contains a large amount of methacrylate groups, which are rich in oxygen; thus, O=C-O, C-O, and C=O tended to increase gradually in treatments with MPS. According to the O1s spectra, C-O increased up to 10 s of treatment time but decreased at 15 s as a result of resizing with APS (Figure 4b). This means that O=C-O reacted with -SiOH contained in APS to become C-O and C=O, and C-O increased up to 10 s of treatment time. After 10 s, APS actively reacted with both O=C-O and C-O, and C-O decreased. 

Further, the C-O of recycled carbon fiber treated with MPS gradually increased until the treatment time of 15 s (Figure 4e). This is because MPS has a large amount of oxygen. Thus, C-O steadily increased as the processing time increased. According to the Si2p spectra, the Si-O-Si of APS tended to increase continuously as the treatment time increased, which this study attributed to the binding of -SiOH contained in the sizing agent during the resizing process to remove H_2_O. MPS showed the same trend, with Si-O-Si gradually increasing with treatment time, which is thought to be due to the active reaction between -SiOH (Figure 4c,f).

Previous studies have reported that after sizing treatment with DMHM (N-(4′4-diaminodiphenyl methane)-2-hydroxypropyl methacrylate), vinyl functional groups (-CH=CH_2_) are introduced to the carbon fiber surface, increasing the radial width of the C=C peak, and O=C-N bonds are formed by the reaction of COOH (carboxyl group) and NH_2_ (amino group) at the carbon fiber surface [35,36]. Furthermore, sizing with polydopamine can increase the amount of carbon and nitrogen, and C-N bonds have been shown to be generated through the spontaneous oxidative polymerization of dopamine [37]. When treated with poly(phthalazinone ether ketone), bonds such as C-N and C=N appeared due to the formation of phthalazine rings [21], and when an amino–silane coupling agent is used, the content of carbon increases as the length of the chain increases; the Si content also increases as the Si-O-Si bond increases, but the content of Si is thought to decrease because excessively long chains cover the Si located inside [21]. Vinyl ester treated with the R806 sizing agent contains many C-O bonds, which is expected because vinyl ester is the product of an unsaturated monoacid reaction with epoxy [38]. Further, with MR13006, a sizing agent with fewer C-O and more O-C=O than R806, it was difficult to distribute the agent uniformly on the carbon fiber, and it was reported that C-O has better compatibility with the carbon fiber surface than O=C-O [38]. 

In this study, C-O and C=O increased due to the dehydration–condensation reaction of O=C-O and -SiOH in the sizing agent on the surface of recycled carbon fiber, up to the optimum concentration of 1 wt.% and the treatment time of 10 s for APS, and Si-O-Si increased slightly due to the bonding of -SiOH on the surface of carbon fiber. When the treatment time was more than 10 s, C-O and C=O decreased due to the combination of APS, and Si-O-Si increased significantly due to the active reaction of -SiOH on the surface of the recycled carbon fiber. In the case of MPS, at the optimum concentration of 0.5 wt.%, C-O and C=O continuously increased with increasing treatment time due to the methacrylate group present at the end of MPS, and Si-O-Si increased significantly due to the reaction of -SiOH on the surface of recycled carbon fiber. Plausibly, these oxygen functional groups increase the surface energy of the recycled carbon fiber and improve the interfacial bonding.

**Figure 5:**
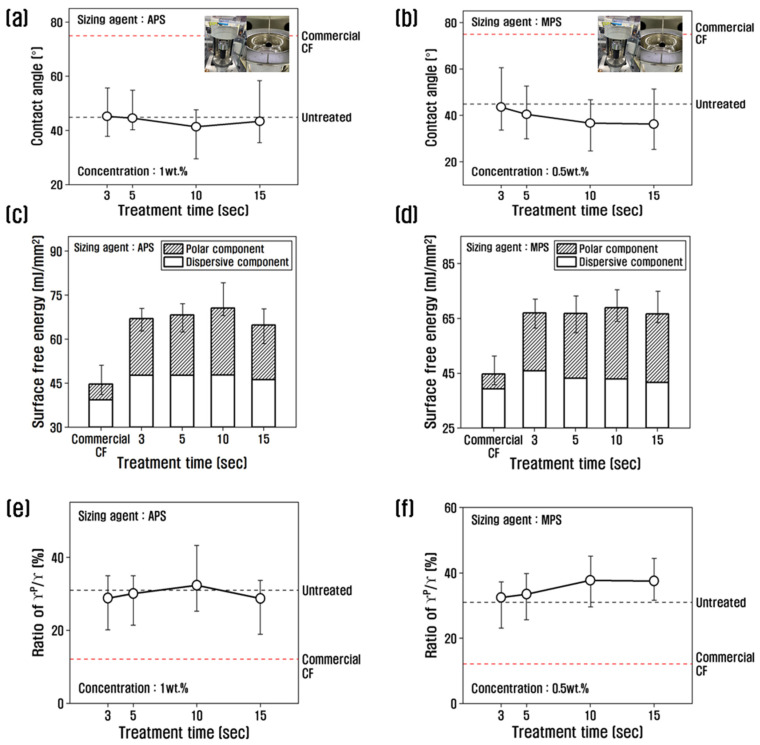
Variation of contact angle, surface free energy, and γ^p^/γ of recycled carbon fibers treated with (**a**,**c**,**e**) APS and (**b**,**d**,**f**) MPS.

To investigate the changes in surface free energy of recycled carbon fibers due to the resizing process, the contact angle was measured, and the values were substituted into the following Equation (1) [39] to calculate the polarized and non-polarized surface free energy, which is shown in Figure 5.

(1) $$\frac{\gamma_{L}(1+\cos\theta )}{2{\left(\gamma_{L}^{D}\right)}^{\frac{1}{2}}}={\left(\gamma_{S}^{P}\right)}^{\frac{1}{2}}\times {\left(\frac{\gamma_{L}^{P}}{\gamma_{L}^{D}}\right)}^{\frac{1}{2}}+{\left(\gamma_{S}^{D}\right)}^{\frac{1}{2}}\;$$

With APS at 1 wt.%, the contact angle decreased up to 10 s of treatment time, reaching the lowest value of about 41.36°, and then increased slightly at 15 s. The contact angle of carbon fiber treated with MPS at a concentration of 0.5 wt.% decreased until 10 s of treatment time. By contrast, the carbon fiber treated with MPS at a concentration of 0.5 wt.% showed a decreasing trend up to 10 s and almost no change beyond 10 s. From the contact angle results, the surface energy as a function of the resizing treatment time showed that the surface energy of the recycled carbon fiber treated with 1 wt.% APS increased up to 10 s of treatment time and decreased beyond 10 s. The increase was due to the polar surface area. The increase up to 10 s was due to the increase in polar surface energy, while the non-polar surface energy did not change. The polarity/surface energy ratio showed the highest value of 32.29% at 10 s, which is about 2.6 times higher than that of commercial carbon fiber, and decreased at treatment times above 10 s, compared to 10 s. It is possible that when APS is coated on recycled carbon fibers at 1% or more, it breaks the oxygen functional groups and actively binds the APS, reducing the amount of oxygen and thus reducing the polar surface energy. 

When treated with MPS, the polar surface energy was the highest at 10 s; the change was insignificant after 10 s and the polar/surface free energy ratio was about 38% at 10 s, which is about 3 times higher than that of commercial carbon fiber. This is believed to be due to the large amount of oxygen contained in MPS, which greatly enhances polar surface energy. In previous studies, the polar surface free energy was shown to increase due to -NH_2_ after treatment with poly(amidoamine), and it increased continuously with increasing concentration [34]. Sizing agents can also increase the amount of oxygen functional groups, such as C=O, in carbon fibers to improve the polar surface energy [18].

### 3.4. Mechanism of Functional Group Change during Resizing Treatment

**Figure 6:**
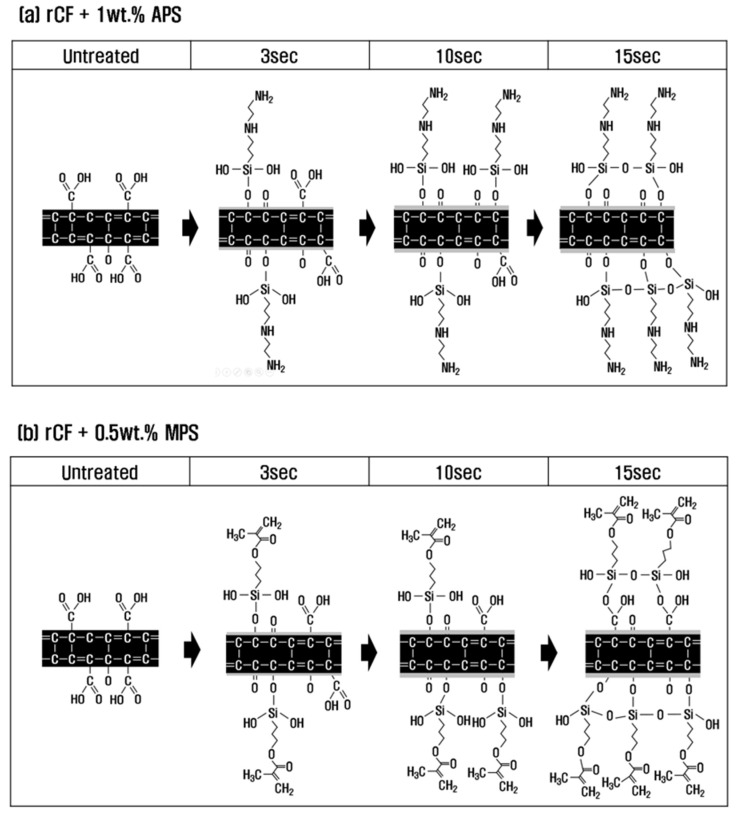
Schematic of the chemical reaction of carbon fibers according to sizing treatment time. (**a**) rCF/APS; (**b**) rCF/MPS.

To investigate the effect and mechanism of PA6 and PP silane coupling agents on the interfacial bonding force between recycled carbon fibers and thermoplastics, surface treatment with nitric acid was performed under optimal conditions, followed by sizing treatment. Based on the results of analyzing the mechanical and chemical properties of recycled carbon fibers according to the concentration of the sizing agent and treatment time, the chemical structure and functional group mechanisms of recycled carbon fibers are shown in Figure 6.

With a PA6-based 1 wt.% APS sizing agent, H_2_O was removed by a dehydration–condensation reaction between O=C-O present on the surface of recycled carbon fiber and -SiOH contained in APS at a treatment time of 3 s (Equations (2) and (3)). At this time, O=C-O was converted into C-O and C=O by breaking the bonds between carbon and oxygen, and it seems that O=C-O gradually decreased and C-O and C=O increased slightly. In general, sizing agents react with water to undergo hydrolysis, in which -OCH_3_ (methyl groups) become -OH (hydroxyl groups), and undergo a dehydration–condensation reaction with the oxygen functional groups of recycled carbon fibers [40,41]. In this study, H_2_O was removed, and O=C-O was converted to C-O and C=O through a dehydration–condensation reaction between O=C-O present on the carbon fiber surface and -SiOH contained in APS. At 10 s, similar to the trend at 3 s, O=C-O decreased, and C-O and C=O continued to increase, suggesting that -SiOH mainly reacted with O=C-O at this time. Thus, this study can conclude that at a treatment time of 10 s, C-O and C=O increased to the maximum, and the interfacial bonding force was maximized due to the optimal oxygen to carbon ratio (O/C). When the treatment time was 15 s or more, O=C-O, C-O, and C=O were all reduced due to the dehydration–condensation reaction with the -SiOH contained in the sizing agent, which broke the bond between carbon and oxygen. Si-O-Si was greatly increased by reacting with each other and combining with the -SiOH contained in the sizing agent during the resizing treatment. As a result, O/C decreased due to the decrease in C-O and C=O, and the interfacial binding force decreased somewhat more than at the treatment time of 10 s.

In the case of the resizing treatment with 0.5 wt.% of PP-based MPS sizing agent, H_2_O was removed by a dehydration–condensation reaction of O=C-O on the carbon fiber surface with the -SiOH contained in the sizing agent at a treatment time of 3 s. At this time, the bond between the carbon and oxygen of O=C-O was broken, and the methacrylate group (H_2_C=C(CH_3_)CO_2_H) containing a large amount of oxygen from the MPS was introduced into the broken bond, and O=C-O, C-O, and C=O increased (Equations (2)–(4)). At a treatment time 10 s, similar to the trend at 3 s, O=C-O and the -SiOH contained in the sizing agent underwent a dehydration–condensation reaction. The bonds of O=C-O were broken, and the methacrylate group was continuously and significantly introduced into the broken bonds, and O=C-O, C-O, and C=O continued to increase. For up to 10 s of treatment time, SiOH was judged to have reacted mainly with O=C-O. As a result, at 10 s of treatment time, the optimum O/C was obtained due to an increase in O=C-O, C-O, and C=O, which represents the maximum interfacial bonding force. At 15 s or more, not only O=C-O but also C-O and C=O present on the surface of the carbon fiber underwent a dehydration–condensation reaction with the -SiOH contained in the sizing agent, breaking the bond between carbon and oxygen but greatly introducing methacrylate groups containing a large amount of oxygen, resulting in a significant increase in C-O, C=O, and Si-O-Si. In addition, at a treatment time of 15 s or more, the -SiOH contained in the sizing agent reacted and bonded with each other, resulting in an increase in Si-O-Si. As a result, C-O and C=O continued to increase at 15 s rather than at 10 s, but the interfacial bonding force decreased within the error range. Thus, this study judged that optimal sizing was achieved at the shortest time of 10 s.
O=C−O↓+−SiOH $↗C=O↑+C-O-Si+H_{2}O\;$(2)$↘C-O↑+C-O-Si+H_{2}O\;$(3)−SiOH+−SiOH→$Si-O-Si+H_{2}O\;$(4)

## 4. Conclusions

In this study, the thermal, mechanical, and chemical properties of recycled carbon fibers were analyzed according to the sizing agent concentration and treatment time after desizing, the optimal conditions were derived, and the chemical changes and functional group mechanisms according to the desizing treatment conditions were identified.

1. In the case of resizing with the PA6-based APS sizing agent, at a concentration of 1 wt.% of the sizing agent and a treatment time of 10 s, the O=C-O present on the surface of the carbon fiber and the -SiOH contained in the APS underwent a dehydration–condensation reaction, and the O=C-O was converted into the bonds of C-O and C=O, the C-O and C=O increased, and from this, the effect of C-O and C=O on the interfacial bonding force increased to the maximum, and the ratio between oxygen and carbon (O/C) was 0.26. In addition, the polar/surface energy ratio showed the highest value of 32.29% at 10 s, and the interfacial bonding force showed the maximum value of 32 MPa at 10 s, which is about 15% better than that of commercial carbon fiber, and was determined to be the optimal PA6-based sizing condition.
2. When the PP-based MPS sizing agent was used, both C-O and C=O as well as O=C-O, at a concentration of 0.5 wt.% and treatment time 10 s, were subjected to a dehydration–condensation reaction with -SiOH, which broke the bonds between carbon and oxygen and introduced oxygen-rich methacrylate groups (H_2_C=C(CH_3_)CO_2_H) into the broken bonds, resulting in a significant increase in C-O and C=O and a significant increase in O/C to 0.51. Further, this study recorded a polar/surface free energy ratio of about 38% at 10 s, and the interfacial bonding force maximally increased to about 27%, compared to commercial carbon fiber, which was judged to be the optimal PP-based sizing condition.

The interfacial shear strength characteristics reported in this study are comparable to those of commercial carbon fiber, which are typically coated with epoxy sizing agents. This outcome is attributed to the resizing of the carbon fibers in this study, achieved by selecting sizing agents that had excellent chemical coordination with each resin after surface treatment with nitric acid, an optimal condition indicated in previous studies [37]. In the future, this study plans to produce carbon composites by impregnating recycled carbon fibers and thermoplastic resins under optimal conditions to evaluate whether their mechanical properties are equivalent to those of commercial carbon composites, which can contribute to the commercialization of automotive parts built with these materials.

## Figures and Tables

**Table 1:** Properties of carbon fiber in this study.

Type	Commercial CF	Recycled CF
Tensile strength (Gpa)	4.49	3.45
Modulus (Gpa)	261	256
Elongation (%)	2.62	2.08
Density (g/cm^3^)	1.80	1.80

**Table 2:** Surface element composition of recycled carbon fibers according to sizing treatment conditions.

Treatment Condition		Elemental Composition (at. %)				O/C
Sizing Agent	Time (s)	Carbon	Oxygen	Nitrogen	Silicon	
Commercial CF		76.31	21.31	0.75	1.63	0.28
Untreated		73.91	22.81	2.73	0.55	0.31
APS (1 wt.%)	3	63.79	13.86	13.44	8.91	0.22
	5	62.71	14.28	13.73	9.28	0.23
	10	60.61	15.59	14.11	9.69	0.26
	15	62.54	12.56	14.77	10.13	0.20
MPS (0.5 wt.%)	3	64.08	27.51	0.27	8.14	0.43
	5	60.64	29.42	0.32	9.62	0.49
	10	58.55	29.80	0.30	11.35	0.51
	15	55.20	31.28	0.28	13.24	0.57

**Table 3:** Functional group according to sizing agent and treatment time by XPS.

Treatment Condition		C1s (at. %)			
Sizing Agent	Time (s)	C-C, C=C	C-O, C=O	C-N	O=C-O
Commercial CF		71.09	26.86	0.98	1.07
Untreated		74.00	8.12	6.28	11.59
APS (1 wt.%)	3	60.75	20.04	11.84	7.31
	5	60.13	20.83	12.91	6.13
	10	60.06	22.19	13.74	4.01
	15	63.19	19.95	14.27	2.59
MPS (0.5 wt.%)	3	76.05	10.53	5.27	8.15
	5	71.40	14.36	4.70	9.54
	10	62.43	22.47	3.70	11.40
	15	58.58	24.13	3.21	14.08

## Data Availability

Not applicable.
